# The Integrated mRNA and miRNA Approach Reveals Potential Regulators of Flowering Time in *Arundina graminifolia*

**DOI:** 10.3390/ijms24021699

**Published:** 2023-01-15

**Authors:** Sagheer Ahmad, Chuqiao Lu, Jie Gao, Yonglu Wei, Qi Xie, Jianpeng Jin, Genfa Zhu, Fengxi Yang

**Affiliations:** 1 Guangdong Key Laboratory of Ornamental Plant Germplasm Innovation and Utilization, Environmental Horticulture Research Institute, Guangdong Academy of Agricultural Sciences, Guangzhou 510640, China; 2 Guangdong Laboratory of Lingnan Modern Agriculture, Guangzhou 510640, China

**Keywords:** micro RNA, floral integrators, bamboo orchid, multi-omics, hormones

## Abstract

Orchids are among the most precious flowers in the world. Regulation of flowering time is one of the most important targets to enhance their ornamental value. The beauty of *Arundina graminifolia* is its year-round flowering, although the molecular mechanism of this flowering ability remains masked. Therefore, we performed a comprehensive assessment to integrate transcriptome and miRNA sequencing to disentangle the genetic regulation of flowering in this valuable species. Clustering analyses provided a set of molecular regulators of floral transition and floral morphogenesis. We mined candidate floral homeotic genes, including *FCA*, *FPA*, *GI*, *FT*, *FLC*, *AP2*, *SOC1*, *SVP*, *GI*, *TCP*, and *CO*, which were targeted by a variety of miRNAs. MiR11091 targeted the highest number of genes, including candidate regulators of phase transition and hormonal control. The conserved miR156-miR172 pathway of floral time regulation was evident in our data, and we found important targets of these miRNAs in the transcriptome. Moreover, endogenous hormone levels were determined to decipher the hormonal control of floral buds in *A. graminifolia*. The qRT-PCR analysis of floral and hormonal integrators validated the transcriptome expression. Therefore, miRNA-mediated mining of candidate genes with hormonal regulation forms the basis for comprehending the complex regulatory network of perpetual flowering in precious orchids. The findings of this study can do a great deal to broaden the breeding programs for flowering time manipulation of orchids.

## 1. Introduction

Orchidaceae is one of the largest families of flowering plants, containing about 28,000 species [1,2,3]. Commercially popular orchids, such as *Cymbidium*, *Phalaenopsis* and *Dendrobium*, bloom in specific seasons [4]; however, *Arundina graminifolia* blooms throughout the year. It is commonly called “Bamboo Orchid” and mainly grows in the tropical and sub-tropical areas of Asia [5,6,7,8]. It grows along ravines, under shrubs, on grassy slopes, or in forests (at altitudes between 400 and 2800 m), exhibiting strong adaptability [8]. Considering its short vegetative phase, it is imperative to understand the genetic network underlying floral development in *A. graminifolia* in order to change floral traits and modify flowering time for other orchids. Although much research has been done on the flowering of orchids [9,10,11], the role of microRNAs in the floral development of *A. graminifolia* remains undefined.

Small, non-coding RNAs are generally grouped into short interfering RNAs (siRNAs) and microRNAs (miRNAs). Both classes possess the same chemical composition and mode of action. However, miRNAs and siRNAs differ in their evolutionary conservation, origin and the gene types they target [12]. MiRNAs are non-coding endogenous RNAs of about 21 nucleotides in length. They are derivatives of single-stranded RNA hairpin precursors, which are cleaved by Dicer (double-stranded-specific RNase) in animals and DCL1 (Dicer-like1) in plants [13]. The trimming of the hairpin precursor by DCL1 releases the miRNA/miRNA*, resulting in the quick degradation of most of the miRNA* sequences [14,15]. However, some miRNA* are found in a large abundance, such as two families of conserved miRNA, miR396 and miR171, which were identified in *Brassica napus* [16] and *Rosa hybrida* [17]. In plants, species-specific or non-conserved small interfering RNAs are the trans-acting small interfering RNAs (ta-siRNAs). Ta-siRNAs act in trans to control messenger RNAs (mRNAs) [18]. Ta-siRNAs are derived from TAS gene transcripts cleaved by a miRNA, thus producing small fragments of RNA (21 nt long) that are in phase with the cleavage site of miRNA [19].

MiRNAs play a vital role in floral organ identity and flowering time manipulation. In Arabidopsis, three families of miRNA (miR172, miR159 and miR156/157) have been shown to control flowering time under a conditional environment. Flowering can be delayed significantly by the overexpression of miR156/157 [20]. In short-day photoperiods, miR159 is shown to regulate floral transition by repressing identity genes of floral meristem [21]. Flowering is promoted by miR172, which integrates ambient temperature signals into the genetic network to promote flowering [22]. Similarly, miR156 and miR172 control the phase change of spikelet meristem to floral meristem in maize and rice to trigger the floral organ primordia [23,24]. Both the miRNAs involve floral organ formation, wherein miR172 regulates the formation of inner whorl [25], and miR164 controls the boundary established between components within floral organs [26]. Recently, a few studies have documented the role of miRNAs in some important orchids, such as *Phalaenopsis* [27], *Erycina* [28] and *Orchis* [29], but not in *A. graminifolia*.

Phytohormones play key roles in controlling developmental stages across the life cycle of plants. The distribution of auxin establishes a gradient that promotes organ development. The role of auxin is tightly linked to cytokinin regulation, and both hormones control organ initiation. Gibberellic acid (GA) has an established role in flowering, stem elongation and germination. ABA is the primary antagonist of GA. Abscisic acid (ABA), along with ethylene and jasmonic acid, is also known for stress-induced responses [30]. Based on trans-regulation capabilities and pivotal developmental functions, miRNAs are considered to be excellent candidates for integrating multiple hormonal responses. The miR396 negatively regulates cell division through the downregulation of GRF genes in various plant tissues [31,32,33,34]. The miR393 has been shown to interact with the genes related to phytohormones [34]. MiR160 and miR167 are the active regulators of auxin responses by influencing the expression of ARFs [35,36]. Transgenic plants expressing *AtARF10* (resistant to miR160) showed elevated expression of a number of ABA-related genes, suggesting auxin control of ABA response [30]. The miR156 promotes flowering in Arabidopsis by regulating the GA pathway [37]. In long days, degradation of DELLA proteins through GA results in SPL-mediated activation of floral identity genes and miR172, thereby causing floral initiation [38]. However, detailed studies are required to disentangle the complex network of miRNA-mediated regulation of floral timing through phytohormones.

This study reports the integrated loop of transcriptome, miRNA and Degradome sequencing to decipher the complex phenomenon of flower timing in *A. graminifolia*. The short *A. graminifolia* sequences were searched to forecast conserved and novel miRNAs. *A. graminifolia* mRNA transcriptome was created to explore potential genes targeted by miRNA. The specific outcomes will form the basis for discovering miRNAs and their respective targets involved in *A. graminifolia* flower development and floral time regulation.

## 2. Results

### 2.1. Continuous Flowering and Flower Characteristics of A. graminifolia

*A. graminifolia* gains reproductive maturity six months after transplantation from a culture medium. However, other orchids need a vegetative phase of 2–3 years, such as *Cymbidium*, *Phalaenopsis*, *Oncidium* and *Dendrobium*. *A. graminifolia* blooms throughout the year, with peak flowering from September to January. Its inflorescence stalk is called a raceme that bears an average of 6.1 flowers. A single flower lasts for 32.3 days; however, the ornamental span of flowering may last up to five months (Supplementary Figure S1).

The flower develops in six stages (stages 0–5). Inflorescence meristem (IM) develops in stage 0 and has flattened primordium (Supplementary Figure S2). IM produces a floral structure on the shoot apex, and the floral primordia divide into separate primordia for the sepal, petal, labellum, and column. Compared with other orchids, this process goes much faster in *A. graminifolia*, completed in 2–3 days. In the second stage, the floral organs start to differentiate, establishing a zygomorphy typical of orchid flowers (Supplementary Figure S2C). In the third stage, the floral tip looks like an inverted angle, wherein the outer sepals overlap with the petals on the inner side (Supplementary Figure S2F,G), while the labellum forms a kinked to undulated margin (Supplementary Figure S2D–G). Gynostemium elongates in stage four, and the labellum gets color (Supplementary Figure S2H–K). Finally, the pollinia mature in stage five, and the flower opens, bearing a fine-structured column in the center (Supplementary Figure S2L–O) and four pollinia arranged on a semi-circular viscidium (Supplementary Figure S2N).

### 2.2. Transcriptome-miRNA Sequencing and Functional Annotation

**Figure 1:**
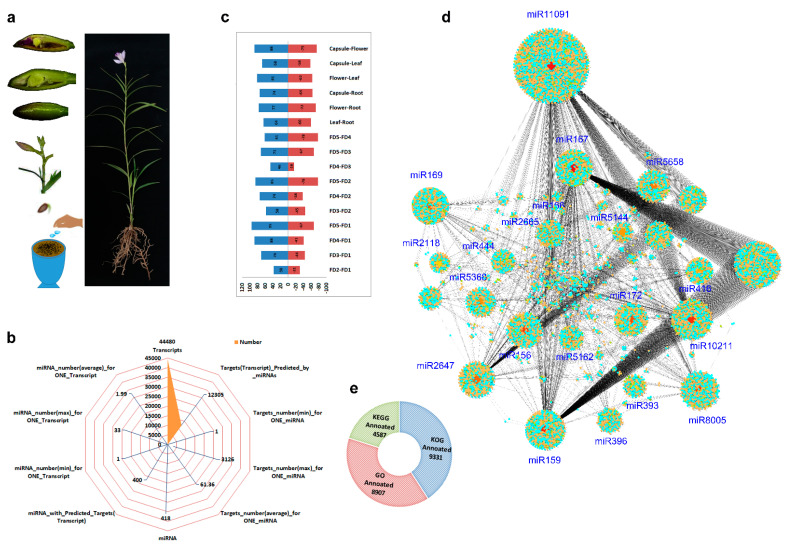
The process of plant production and material selection for sequencing (**a**); the miRNA distribution according to mRNA targets (**b**); comparison of up- and down-regulated miRNAs among nine samples (blue bars show upregulation and red bars show downregulation)(**c**); overall distribution of miRNAs along with their predicted mRNA targets (blue target color shows upregulated and orange color shows downregulated genes) (**d**); and the annotation statistics of mRNA for KEGG, KOG and GO annotations (**e**).

The plants were generated from seeds, and after six months, fully developed plants were used for transcriptome analysis (Figure 1a). Transcriptome analysis and subsequent target prediction by miRNA generated system-level clues about the mechanism that controls flower initiation and development in *A. graminifolia*. The transcriptome data produced 71.2 billion high-quality reads from all the samples, with each sample producing about 10.8–12.8 Gb of data (~7.8 billion reads).

We filtered 44,480 transcripts, of which 12,305 transcripts were identified as targets for miRNA (Figure 1b). A total of 418 miRNAs were identified, and 400 miRNAs possessed targets (transcripts). The maximum number of targets for one miRNA was 3126, while the maximum number of miRNAs for one transcript was 33 (Figure 1b). We compared the samples in 16 pairs and identified miRNAs with opposite expression intensities between various combinations (Figure 1c). The comparison between capsule-mature flower, FD2–FD5 and FD1–FD5 yielded the highest number of miRNAs with antagonistic expressions (Figure 1c). The lowest numbers of antagonistic miRNAs were observed between FD3 and FD4. An overall clustering highlighted the candidate miRNAs along with their predicted targets (Figure 1d). The major clusters were produced by miRNAs related to flowering and hormone regulation, such as miR159, miR172, miR156 and miR396. The miR11091 showed the highest number of targets.

The genes targeted by miRNAs were annotated using GO, KEGG and KOG databases. The annotation produced 9331, 8907 and 4587 in KOG, GO and KEGG categories, respectively (Figure 1e). For flower development, auxin-activated signaling and anther development were the abundant GO terms in miRNAs, while plant hormone signal transduction was the most abundant pathway in KEGG enrichment (Supplementary Figure S3a). On a minor level, seed development, leaf development, floral organ senescence and maintenance of shoot apical meristem were the GO biological process categories relating to plant development (Supplementary Figure S3b). Other than plant hormone signal transduction, a number of KEGG-annotated genes were related to the circadian rhythm in plants, protein export and photosynthesis (Supplementary Figure S3c).

Conserved miRNA families were found abundant in the microRNA data. Some groups were related to flower development, floral whorl development, petal development, sepal development and meristem determinacy (Supplementary Figure S4). Conserved miRNA ids were laid on Cytoscape, and important clusters were found related to flower development and ABA signaling (Supplementary Figure S5). *Glycine max* miRNAs were the most abundant among miRNA families, followed by miRNAs from *Malus domestica*, *Populus trichocarpa* and *Zea mays* (Supplementary Figure S6).

### 2.3. One miRNA with the Highest Targets Responds to Multi-Level Regulation

**Figure 2:**
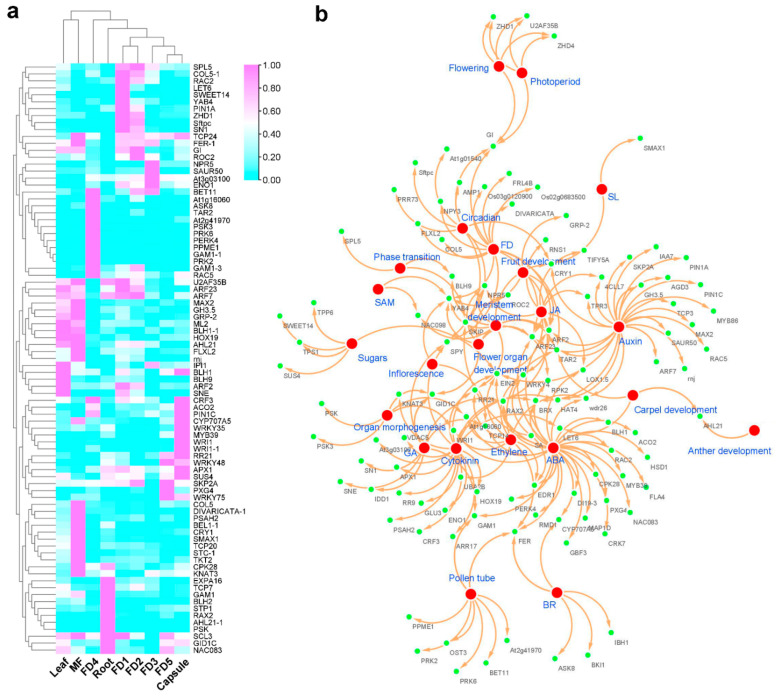
miRNA with the highest mRNA targets. (**a**) heatmap of all the important genes targeted by miR11091; (**b**) clustering analysis of biological processes regulated by miR11091-mediated transcripts.

The lja-MIR11091 was the most abundant miRNA among all the miRNAs identified in our study. It targeted 3126 transcripts and involved multiple processes related to hormonal regulation and flower development. Based on the biological process annotation of the miR11091 targets, we identified important gene families (Figure 2a). More genes were upregulated in mature flowers compared to other tissues, while the most downregulated genes were observed in FD3. Moreover, we divided all the important gene targets of miR11091 into different categories according to their GO biological processes (Figure 2b). The highest number of targets were related to ABA biosynthesis and signaling (31 targets). Auxin control was the second most abundant biological process shown by 23 transcripts. The miR11091 targeted some well-known floral and hormonal regulators, including genes related to ABA regulation (*CYP707A5*, *GAM1*), auxin homeostasis (*ARFs*, *PINs*), cytokinin homeostasis (*ARR17*, *CRF3*), GA regulation (*GID1C*), *CONSTANS*-like (*COLs*), circadian clock (*GI*), vegetative to a reproductive phase change (*SPL5*), flower development (*SPY*, *YAB4*), floral timing (*NAC098*) and sugar homeostasis (*SUS4*, *SWEET14*). However, most of the genes showed multiple regulatory roles (Figure 2b).

### 2.4. Major miRNA Classes Were Found for Flower Bud Control

**Figure 3:**
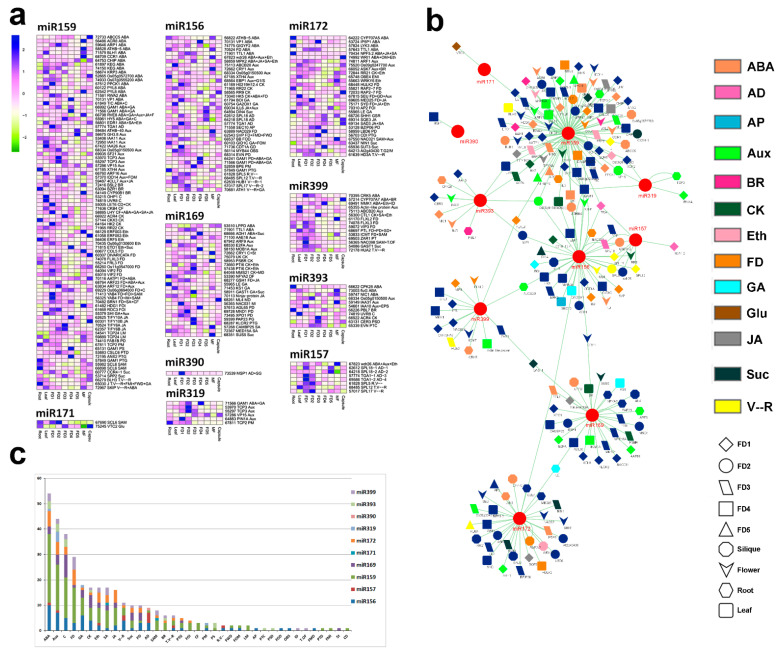
Major miRNA families for flower regulation. (**a**) heatmap of genes targeted by each miRNA family; (**b**) gene distribution for each miRNA family, color bands show biological process enrichment of target genes, node shapes show the stage-specific high expression of target genes; (**c**) relative abundance of biological process shown by all gene targets of miRNA families (ABA: Abscisic Acid; Aux: Auxin; C: Carpel; FD: Flower Development; GA: Gibberellic acid; CK: Cytokinin; Eth: Ethylene; SA: Salicylic Acid; JA: Jasmonic Acid; V—R: Vegetative to Reproductive phase change; Suc: Sucrose; PD: Pollen Development; AD: Anther Development; SAM: Shoot Apical Meristem; BR: Brassinosteroid; T.V--R: Timing of Vegetative to Reproductive phase change; PTG: Pollen Tube Growth; FOI: Floral Organ Initiation; CF: Carpel Formation; PM: Pollen Maturity; PS: Pollen Sterility; R.V--: Rate of Vegetative phase change; FWD: Floral Whorl Development; FOM: Floral Organ Maturation; AP: Acceptance of Pollen; PTC: Pollen Tube Reception; PSD: Pollen Sperm Cell Differentiation; FOD: Flower Organ Development; Organ Boundary Specification; ID: Inflorescence Determination; T.OF: Timing of Organ Formation; FMD: Floral Meristem Development; FMI: Floral Meristem Identity; St: Strigolactone; CD: Carpel Development).

Some of the miRNAs known for flower time control were found in our data, including miR156, miR157, miR159, miR169, miR171, miR172, miR319, miR90, miR393 and miR399. The transcripts targeted by these miRNAs are shown in Figure 3a with their expression intensities. The miR159 contained the highest number of targets among the other miRNAs. Most of its targets were related to ABA biosynthesis and signaling, followed by auxin homeostasis. Targets related to GA signaling and flower development were also predicted by miR159. *BLH3* is involved in the control of phase change from the vegetative to the reproductive stage. A MADS-box transcription factor (J) that controls the timing of vegetative stage initiation, flower meristem identity, flower whorl development and GA signaling is also targeted by miR159.

The role of miR156 is well established in floral control in model crops like Arabidopsis. We found important transcriptomic targets for miR156. It targets *SVP*, which is a major gene for floral bud control. *GA20X1* and *GID1C* are major genes in the GA pathway. Moreover, *SPLs* are important regulators of phase transition from the vegetative to the reproductive stage. Therefore, miR156 pointed out important targets involved in flower development and flowering time.

MiR157 was mainly involved in controlling the genes responsible for anther development (*SPL18* and *TGA1*) and timing of phase change from vegetative to the reproductive stage (*SPLs*). The miR169 pointed out important regulators of hormones and pollen development. Auxin signaling gene (*ARF9*), MADS-box gene (*AGL65*) and sucrose signaling gene (*SUS5*) are important targets for miR169. The miR171 showed only two targets relating to shoot apical meristem (*SCL6*) and glucose (*VTC2*).

The miR172 is well known for floral organogenesis and flowering time control. We found miR172 targets for ABA signaling (*CYP707A5*) and flower development (*RAP2*). *AP2* plays a pivotal role in flower initiation, and *HD3A* is an analog of *FT1* and controls the timing of phase change from the vegetative to the reproductive stage. Both are targeted by miR172.

The miR319 targets genes responsible for hormonal regulation and TCP transcription factors. Only one main target was identified for miR390. It can control anther development through MSP1. The miR393 also regulates major plant hormones, including ABA and auxins. The miR399 targets the genes responsible for flower development, shoot apical meristem and timing of phase change.

Considering the overall target cascade, it can be seen that most of the genes are involved in hormonal regulation compared to other biological processes (Figure 3b). However, some of the genes were targeted by more than one miRNA, and we highlighted those genes with similar color bands (Figure 3b). *TGA1* was targeted by three major miRNAs (miR156, miR157 and miR159). Similarly, *GAM1* was targeted by miR156, miR159 and miR319.

Overall summary of biological process enrichment showed that ABA was the most abundant biological process shown by miRNA targets, followed by auxin signaling. Circadian rhythm was the third most abundant biological process, followed by flower development and GA signaling (Figure 3c).

### 2.5. Stage-Specific miRNAs during Bud Outgrowth

**Figure 4:**
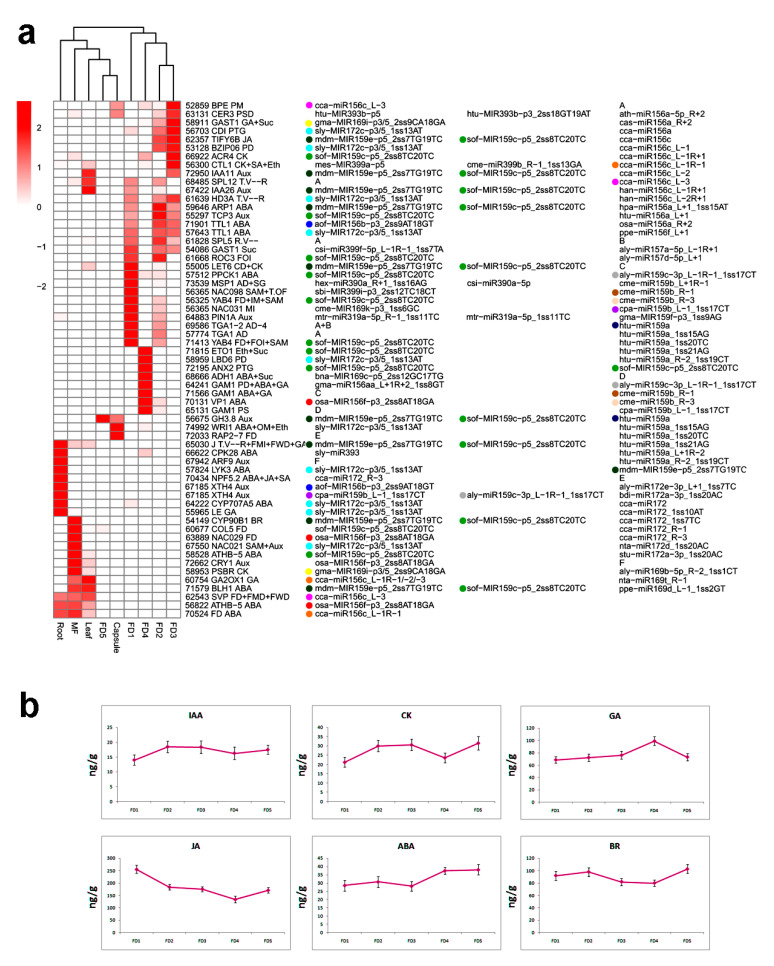
Tissue-specific expression of miRNA targets and hormone concentrations. (**a**) clustering of miRNA targets with their maximum expressions to a particular tissue type. Colored circles show the occurrence of same miRNA for multiple targets (row names show the gene ID, common name and biological process abbreviation, the full abbreviation can be seen in the legend of Figure 3c) (some genes have multiple targets, which are placed separate under headings A-F); (**b**) relative concentrations of different plant hormones in five stages of flower development.

The miRNA targets with the highest expression for a specific developmental stage or tissue type were clustered to predict the specific role of miRNAs (Figure 4a). The highest number of stage-specific miRNA targets was shown by FD1. It included prominent regulators of floral bud initiation, including transcripts related to ABA (*PPCK1*), meristem identity (*YAB4*), anther development (*TGA1*) and carpel development (*LET6*). FD4 exhibited a different group of miRNA targets relating to ABA, GA and pollen development. Here the genes were targeted by miR156, miR159, miR169 and miR172. Like FD4, the miRNA targets were highly expressed in root and mainly related to ABA, GA and auxin.

Interestingly, FD5 showed a response only for *GH3.8*, an auxin signaling gene, and capsule exhibited genes corresponding to flower development (*RAP2*) and ABA signaling targeted by miR172. FD2 mainly responded for *TCP3* (auxin-activated signaling pathway), *TTL1* (ABA signaling), *SPL5* (phase change from the vegetative to the reproductive stage) and *ROC3* (flower organ identity) genes targeted by miR159, miR172, miR156 and miR159, respectively. *HD3A* is an important timer of phase transition, and it was expressed mainly at FD3. The other genes expressed at this stage included genes for auxin, jasmonic acid, cytokinins signaling and pollen tube growth.

### 2.6. Hormonal Regulation Plays a Key Role in Flower Development

Hormones play important roles in the regulation of continuous flowering [39]. We mined a number of miRNA targets involved in the regulation of important hormones. *ARF9*, a candidate gene involved in auxin activated signaling pathway (GO:0009734), was expressed only in roots. *PIN1A*, responsible for polar auxin transport (GO:0009926) and auxin homeostasis (GO:0010252), was expressed in FD1 (Figure 4a). Both are targeted by miR169 and miR319, respectively. Auxin interacts with cytokinin to control organogenesis. LOG1, targeted by miR9773, is an important gene involved in the cytokinin biosynthesis process (GO:0009691). It was expressed mainly at FD5.

Gibberellins are very important in flower development. *GAOX1* regulates the gibberellin catabolic process (GO:0045487) and expresses in leaf and mature flower. It is targeted by miR156. *LE* is a gibberellin biosynthesis gene (GO:0009686). Targeted by miR172, it is expressed only in roots.

Abscisic acid negatively regulates plant organogenesis. We identified targets of miR156 (*FD*, *TTL1*, *VP1*), miR172 (*CYP707A5*, *LYK3*) and miR159 (*PPCK1*) in ABA biosynthesis and signaling. *CYP707A* involves ABA metabolic process (GO:0009687) and is expressed only in roots.

To validate the effect of hormones on orchid flower development, we determined the concentrations of endogenous hormones—abscisic acid (ABA), indole acetic acid (IAA), cytokinins (CK), gibberellic acid (GA), jasmonic acid (JA) and brassinosteroid (BR)—at five stages of floral development (Figure 4b). IAA showed an increasing trend from FD1-FD3 and exhibited lower concentrations in FD4 and FD5. The same trend was shown by cytokinins; however, the overall cytokinin concentration was higher than IAA in all stages of floral development. Contrarily, JA showed decreasing trends from stage FD1 to FD4 and then increased in FD5.

ABA and GA showed the highest concentrations in FD4; however, GA contents were much higher in FD4 as compared to BA. Brassinosteroid exhibited higher concentrations in FD2 and FD5 compared to the rest of the stages (Figure 4b).

### 2.7. qRT-PCR of Selected Flowering Regulators

**Figure 5:**
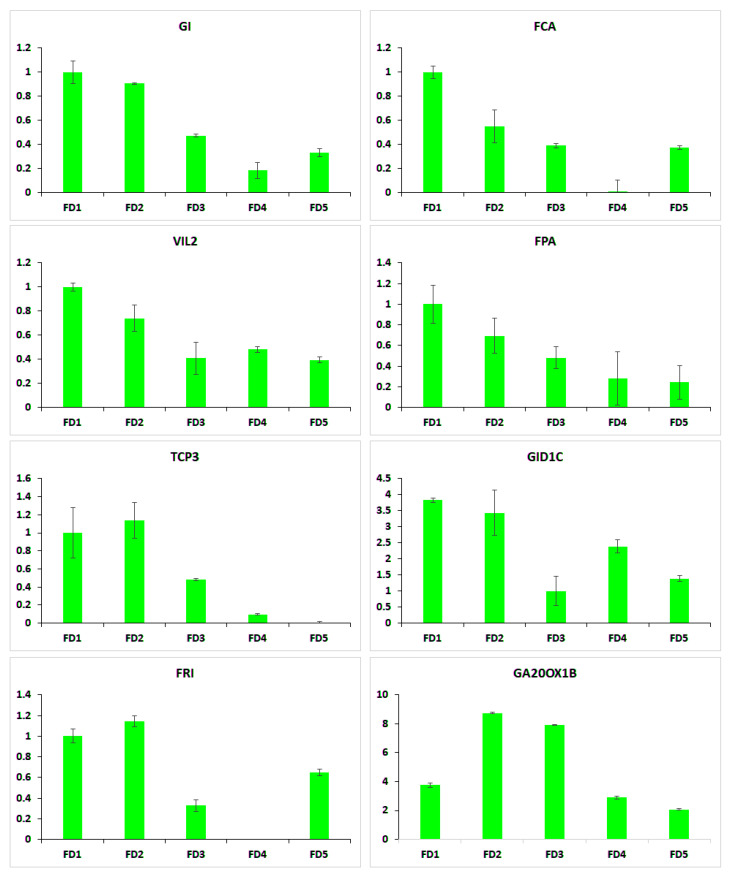
The qRT-PCR analysis of selected candidate mRNAs.

Eight candidate flowering regulator genes were selected to be validated through qRT-PCR (Figure 5). These included flowering time regulators *FCA*, *FPA* and *GI*, flower development genes *VIL2*, *FRI* and *TCP3* and gibberellin biosynthesis genes *GID1C* and *GA20OX1B*. All these genes showed high expression in the early stages of flower development compared to the late stages. Flowering time genes *FCA*, *FPA* and *GI*, and vernalization pathway gene *VIL2* were specifically expressed in FD1 compared to the rest of the stages.

## 3. Discussion

The beauty of *Arundina graminifolia*, among other orchids, is its perpetual flowering habit. Micro-RNAs play important roles in the regulation of floral meristem identity and floral timing in model plants. However, the molecular regulation of continuous flowering and floral timing remains poorly defined in *A. graminifolia*. To extend our understanding of continuous flowering, we integrated de novo RNA-seq, miRNA and Degradome target prediction to study the molecular dynamics of flower development. Several miRNA targets were expressed during the developmental stages of the floral bud. The comprehensive analysis indicated miRNAs specific to pivotal genetic and hormonal regulators of flower development (Supplementary Table S1).

Intricate networks are involved in controlling multiple signaling pathways for flower development [40]. The transcriptomic data pointed out important floral regulators, such as *COLs*, *ELFs*, *SOC1*, *GI* and *FT* (Figure 2b). In addition, *FRIGIDA* is an important regulator of vernalization [41]. It was expressed in the early stages of development and leaf. Moreover, homologs of DELLA proteins (GAI), GID1C, LE and GA20ox1B in the GA pathway and homologs of *FPA* and *FCA* of the autonomous conduit were recognized in this study (Figure 3a). Therefore, the *A. graminifolia* transcriptome possessed a long range of miRNA targets from the five classical pathways of flowering control that have been found in model plants.

The miR11091 targeted the highest number of genes (Figure 2). These targets included important regulators of hormones, such as ABA biosynthesis and signaling (31 targets) and auxin regulation (23 targets). The key targets included ABA regulation genes (*CYP707A5*, *GAM1*), auxin homeostasis genes (*ARFs*, *PINs*), cytokinin homeotic genes (*ARR17*, *CRF3*), GA regulation (*GID1C*), *CONSTANS*-like (*COLs*), circadian clock genes (*GI*), vegetative to a reproductive phase change (*SPL5*), flower development (*SPY*, *YAB4*), floral timing (*NAC098*) and sugar homeostasis (*SUS4*, *SWEET14*) (Figure 2b). This could be interesting because miR11091 has not been discussed previously in flowering regulation. Among the other important miRNAs regulating flower development, miR159 was the most abundant in *A. graminifolia*, followed by miR156 and miR172 (Figure 3a). Previous studies suggest that mtr-miR156a in *Medicago truncatula* [42], zma-miR156a in maize, osa-miR169 in rice and tae-miR169b in wheat [43] are the most abundant miRNAs. The highly expressed miRNAs may regulate essential functions. For example, miR159 regulates flowering in short days [21,44,45]. Most of the miRNAs targeted more than one gene, suggesting these miRNAs control multiple biological functions in plants. Similarly, several miRNAs targeted one gene. This is because miRNAs could be mapped to the same cDNA at multiple sites, and mRNA can undergo cleavage into different fragments [23].

**Figure 6:**
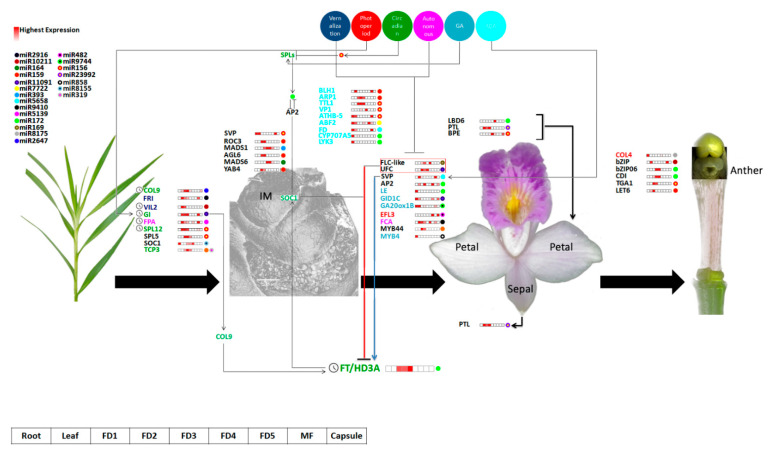
Overview of candidate miRNA targets with their expression intensities and model of miR256-miR172 mediated control of flowering time. The gene colors correspond to pathway colors at the top.

*Squamosa promoter-binding* (*SPB*) genes (*SPL5*, *SPL12* and *TGA1*) showed significant expression in the leaf and the early stages of flower development as compared to other tissues (Figure 6). SPLs are biological clock regulators that regulate phase transition from the vegetative to the reproductive stage [46,47,48]. *SPL12* is targeted by miR156 (Figure 6). As reported previously, miR156 can regulate the SPB gene [49,50]. SPB activates miR172 and MADS-box genes to promote flowering [50]. Overexpression of miR156 blocks the expression of SPB that delays flowering [24]. However, overexpression of miR172 in Arabidopsis accelerates flowering [51,52]. Also, miR172 regulates floral organ identity by targeting the *AP2* gene [25,51]. Therefore, miR156 and miR172 are supposed to act antagonistically in controlling flower development [53,54]. Before entering the vegetative-to-reproductive transition, miR156 decreases in expression, whereas the expression of miR172 increases [51,52,55,56]. The expression of miR172 is also affected by *GIGANTEA* (*GI*), a key circadian clock regulator of the flowering pathway [57]. The expression of miR172 was decreased significantly in the *GI* mutant (gi-2) [58], whereas it was not affected in the co-mutant. These findings suggest that miR172 regulates GI-mediated photoperiodic flowering pathway independent of *CO*. The conserved role of miR156- and 172-mediated phase transition from vegetative to the reproductive stage is documented in *Orchis italica* [28], *Erycina pusilla* [29] and *Phalaenopsis aphrodite* [27]. The *AP2* in our study was targeted by miR172, and it was expressed in leaf, FD2 and FD5 (Figure 3a).

*FLC*, a key flowering repressor, showed high expression in the early stages of flower development along with *UFC* (Upstream of *FLC*) (Figure 6). *FLC* was targeted by miRN169. *FLC* was significantly suppressed in the plants overexpressed with miR169d, resulting in the increased expression of the *FLC*-targeted genes *FT* and *LFY* [59]. The regulatory pathway of miR169 works independently of the miR-156-miR172 pathway [60]. *FRI* induces the expression of *FLC* and negatively regulates flowering [61].

TCP TFs are the targets of miR319 in Arabidopsis [62]. Overexpression of loss-of-function tcp4-1 mutant or miR319 (jaw-D mutant) delayed flowering in Arabidopsis [63,64]. The expression of *TCP* genes was reduced in plants overexpressed with miR319, suggesting a putative role of TCPs in floral time control [59]. Moreover, the upstream region of *SPL3* has conserved *TCP4* binding sites [65], suggesting that *TCP* might directly regulate *SPL3* to control flower time, independent of miR156. However, *TCP4* did not express in any of the tissue types; rather, *TCP3* showed significant expression in the first three stages of flower development, with peak expression in FD2 (Figure 6).

MiR159 targets many *MYB* genes involved in the regulation of flower development [23,66]. It degrades *MYB33* to downregulate *LEAFY* in GA-induced flowering in Arabidopsis, resulting in delayed flowering in short days [21,44,45]. *MYB44* and *MYB4* were targeted by miR159 and miR858, respectively, in *A. graminifolia* and expressed at the root and the early stages of flower development (Figure 6).

The bud’s fate involves integrated roles of endogenous hormones, including growth promoters (IAA, GA, CK and BR) and growth inhibitors (ABA and JA) [67,68,69,70,71,72]. Levels of IAA, GA and BR were higher at FD2 compared to other stages, suggesting a positive effect for rapid growth during the early stages of flower development (Figure 4b). However, ABA concentration was lower in the early stages of flower development as compared to GA, suggesting an antagonistic role of both hormones. ABA controls bud dormancy through the photoperiod pathway [73,74,75] and regulates GA by inhibiting *SVP* in short days. *SVP* upregulates TCP18, which mediates temperature-dependent bud break [76]. Two *SVP* homologs were identified as targets of miR156 and miR5658 (Figure 6). *TCP3* was recognized as the target of miR159 and miR319.

The miR319 and miR396 possess the most molecular connections to the signaling pathways relating to phytohormones [30]. The miR319 positively regulates auxin signaling [77,78], and its overexpression in Arabidopsis and rice affects cell differentiation by inhibiting GA biosynthesis [79,80]. Auxin upregulated miR319 production in *Brassica* while cytokinin repressed its expression in rice, suggesting a critical role of miR319 in coordinating antagonistic auxin-cytokinin pathways [81,82]. Similarly, it could also control antagonistic ABA-GA pathways, as previous studies showed that ABA treatment in rice seedlings inhibited the expression of miR319 [81]. The miR393 plays a role in the unidirectional control of ABA to auxin signaling. ABA upregulates the biosynthesis of miR393 [83] and miR159 [30]. The miR159 constitutes a major link among three hormones—namely ABA, GA and ethylene—to control developmental processes. GA promotes flowering in Arabidopsis through the miR156-mediated pathway [50].

Thus, the vernalization and autonomous pathways repress the function of flowering repressor *FLC*. *FLC* represses *SOC1* and *FT*. The long day photoperiodic pathway upregulates *FT* in the leaves by activating *CONSTANS* (*COL9*) and *GI*. The FT protein moves to the shoot apical meristem through the phloem and induces the meristem identity regulators together with *SOC1* to trigger flowering. The GA pathway crosstalks with the miR156-SPL conduit. The circadian pathway, through differential expression of miR156 and miR172, induces floral integrators through repressing the activity of flowering repressors, thereby turning on the plant’s response towards genetic and environmental signals to start flowering (Figure 6).

Our previous transcriptome mining has elucidated a number of genes that may play crucial roles in the regulation of continuous flowering in bamboo orchids [11,39,84,85,86,87]. Moreover, the enormous medicinal importance of this orchid has also been documented in detail [87]. However, the miRNAs have not been discussed for this unique flowering pattern, especially, the miRNA11091 is a special finding for *A. graminifolia*. This study provides useful information on unique and well-established miRNAs along with their flowering-related targets. This is quite enough to devise various functional studies to manipulate the flowering time and vegetative phase of other orchids.

## 4. Materials and Methods

### 4.1. Plant Preparation and Sampling

*A. graminifolia* plants were grown from seeds at the research facility of Environmental Horticultural Research Institute of Guangdong Academy of Agricultural Sciences, China. The day/night temperature was set to 25/20 °C with a photoperiod of 16/8 h. Samples were obtained from leaves, roots, five stages of flower development (FD1-5), mature flowers and capsules. Sampling was done in three biological and technical repeats for each floral developmental stage and tissue type. A total of 27 plants were used for sampling [39]. After collection in liquid nitrogen, samples were stored at −80 °C until RNA extraction.

### 4.2. Transcriptome Sequencing

- RNA-seq library and sequencing

RNA was extracted using TaKaRa kit, and cDNA libraries were prepared. The mRNAs were filtered using Oligotex mRNA Midi Kit, and cDNA libraries were prepared according to Illumina manufacturing protocol as stated previously [88]. Thus, mRNA fragments (~200 bp) were prepared from total RNA, which were subjected to cDNA synthesis through adapter ligation and low enrichment according to TruSeq^®^RNA HT Sample Prep Kit (Illumina, San Diego, CA, USA). The cDNA was diluted to 10 pM to generate in situ clusters on HiSwq2500 pair-end flow cell, followed by sequencing. Transcriptome de novo was performed with Trinity program using default parameters [89].

#### DEG Analysis

Gene expression of differentially expressed genes was calculated as RPKM value with the following formula:

RPKM = [total exon reads/mapped reads (millions)] × exon length (kb)(1)

The edgeR was used to measure differential expression among tissue types. Threshold *p*-value was determined in various tests using false discovery rate (FDR), and a basic level of significant differential expression was set at FDR < 0.05 and |log2 ratio| > 1 (two-fold change). The correlation between different parts was calculated using Pearson correlation coefficient (PCC). Hierarchical clustering and principal component analysis were performed using prcomp and corrplot utilities of R package [90]. The DEGs were annotated for KEGG and GO enrichment in the databases (http://www.geneontology.org/ or http://www.genome.ad.jp/, accessed on 15 June, 2021) [91,92,93]. A threshold *p*/q-value of ≤0.05 was set to filter significantly enriched terms.

### 4.3. Micro RNA Sequencing

RNA from all tissue types was used to construct small RNA libraries, as described previously [29]. Sequencing was performed using Illumina Hiseq 2500 platform. The output data was filtered, and the reads with Poly N’s and the clean reads (17–30 nucleotides) were mapped to RepeatMasker (www.repeatmasker.org, http://www.genome.ad.jp/, accessed on 15 June 2021), UCSC (gtrnadb.ucsc.edu, http://www.genome.ad.jp/, accessed on 15 June 2021), Rfam (rfam.janelia.org, http://www.genome.ad.jp/, accessed on 15 June 2020) and NONCODE (www.noncode.org/NONCODERv3/download.html, http://www.genome.ad.jp/, accessed on 15 June 2021), to isolate miRNAs from the tags originating from protein-coding genes, repeat sequences, transfer RNA (tRNA), ribosomal RNA (rRNA), small nucleolar RNA (snoRNA) and small nuclear ribonucleic acid RNA (snRNA).

#### 4.3.1. Conserved miRNA Alignment and Expression Analysis

The clean reads were blasted against miRBase using BLASTN. Expression profiles for each miRNA family were ascertained as the sum of all reads annotated to the same family of miRNA in each library, and values were calculated as reads per million (RPM). MiRNAs with *p* < 0.05 and a change ratio of more than 5 or less than 0.5 were regarded as differentially expressed. Statistical analysis was performed following method of Audic and Claverie [94].

#### 4.3.2. Novel miRNA Prediction

Mireap (https://sourceforge.net/projeccts/mireap/, accessed on 16 July 2021) was used to predict novel miRNAs by identifying the Dicer cleavage site and the minimum free energy of the miRNA tags not annotated in the previous steps.

The parameter value -f: (Flank sequence length of miRNA precursor) = 100, and the free energy <−20 kcal mol^−1^ [95].

### 4.4. Degradome Sequencing and Target Prediction

Approximately 20 µg of pooled RNA was used to prepare Degradome library. About 150 ng of poly(A)+ RNA was used as input RNA, which was annealed with biotinylated random primers before streptavidin capture of RNA fragments. The RNAs with 5′-monophosphates were subjected to 5′ adaptor ligation. The clusters were generated using purified cDNA library on Illumina cluster station. Raw reads were analyzed with Illumina Pipeline v1.5, and the extracted reads were used to predict potential cleaved targets with the CleaveLand 3.0 pipeline (http://sites.psu.edu/axtell, accessed on 18 July 2021) [96]. The perfect alignments were kept for degradation analysis. Both Degradome density file and the target finder [94] were used to predict miRNA targets in mRNA.

### 4.5. GO and Pathway Enrichment Analyses

Selective DEGs were clustered for biological processes using Cytoscape with BINGO plug-in [90,95]. For GO terms, the corrected p-value was used as described previously [90].

### 4.6. Hormonal Analysis

Based on the biological processes of the highly differential DEG targets by miRNA, the six major plant hormones were selected to know the hormonal fluctuation at five stages of flower development. The hormonal contents were determined using the HPLC-MS/MS (Agilent) protocol as described by Pan et al. [96].

### 4.7. Scanning Electron Microscopy

Scanning electron microscopy was used to understand the bud initiation from stage 0 to stage 2. Dissected buds were fixed in a solution consisting of 3% glutaraldehyde and 2% formaldehyde for 24 h. The samples were then dehydrated in acetone, followed by drying at a critical point in liquid carbon dioxide. The dried samples were mounted on stubs and sputter coated with 25 nm thick layer of gold. The specimens were observed using a scanning electron microscope (JSM-6360; JEOL).

### 4.8. Quantitative Real Time PCR

The selected flowering and hormone-related genes were validated through qRT-PCR. Five stages of flower development were used to extract total RNA, followed by cDNA synthesis. The reaction mixture contained 20 µL solution, including 10 µL SYBR premix Ex-taq™ (Takara, Kusatsu, Japan). The reaction was performed using Bio-Rad CFX-96 RealTime PCR System (Bio-Rad, Hercules, CA, USA) with *Actin* as internal standard. Three technical replicates were performed. The list of primers is provided in Supplementary Table S2.

### 4.9. Statistical Analysis

SPSS software (SPSS Inc., Chicago, IL, USA; ver. 16.0) was used to analyze the data by One-way ANOVA. Significant differences are shown at *p* < 0.05 or *p* < 0.01 level.

## 5. Conclusions

This study explores the miRNA-mediated genetic protocols for flowering regulation in bamboo orchids (*Arundina graminifolia*). A number of key miRNA targets were predicted through Degradome Target Prediction. These targets mainly included floral time regulators and MADS-box TFs along with hormonal pathway integrators, while hormone fluctuations were also observed among the five stages of flower development. Considering the novelty that *A. graminifolia* completes its vegetative cycle much faster than other orchids, this research further fetches valuable insights to accelerate the breeding programs for short vegetative phase orchids.

## Data Availability

Not applicable.

## Supplementary Materials

The following supporting information can be downloaded at: https://www.mdpi.com/article/10.3390/ijms24021699/s1.
