# Supplementary material for: The Integrated mRNA and miRNA Approach Reveals Potential Regulators of Flowering Time in Arundina graminifolia
Source: Int J Mol Sci. 2023 Jan 15;24(2):1699. doi: 10.3390/ijms24021699 (PMC9865619; doi:10.3390/ijms24021699)

Supplementary Figure S1. Comparison of flowering habit of bamboo orchid with seasonal flowering species.

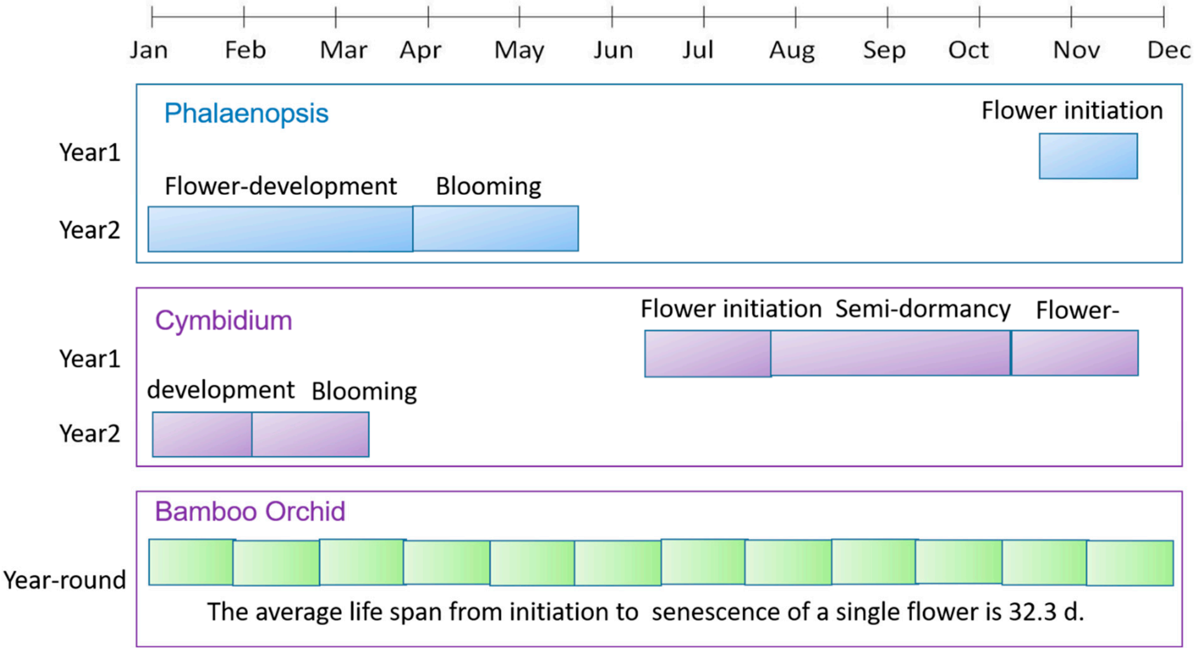

Supplementary Figure S2 Stages of flower development in *Arundina graminifolia*. A: Stage 0 (IM: inflorescence meristem); B: Stage 1 (IM: inflorescence meristem, PP: petal primordia, SP: sepal primordia); C: Stage 2 (PP: petal primordia, SP: sepal primordia, LP: lip primordia, CP: carpel primordia); D-G: Stage 3; H-K: Stage 4; M-O: Stage 5; L: mature flower.

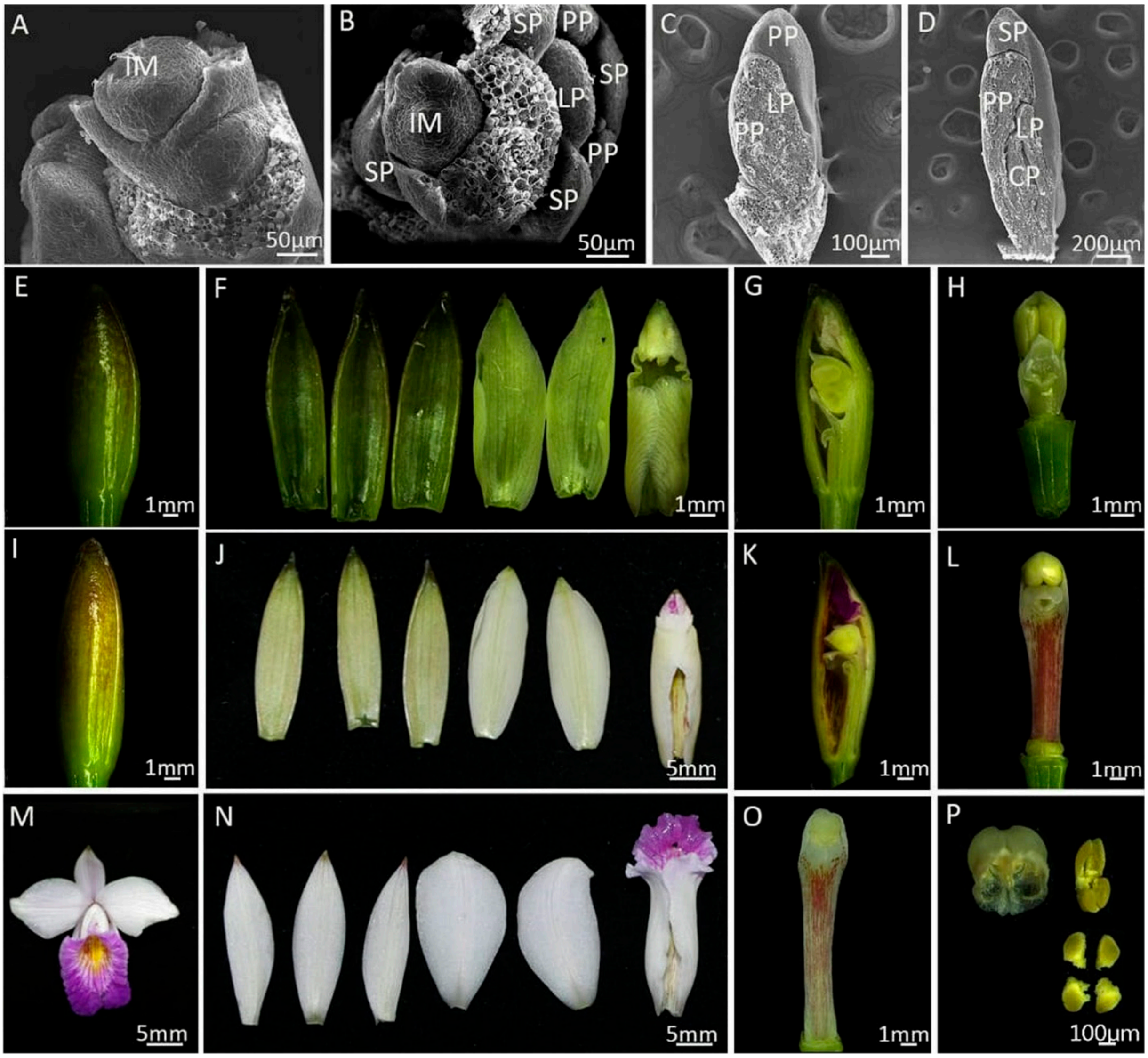

Supplementary Figure S3 GO and KEGG annotation of Degradome sequencing and overview of conserved miRNA families. a: most popular GO and KEGG categories of Degradome sequences; b&c: relative abundance of GO and KEGG enrichment for Degradome sequencing.

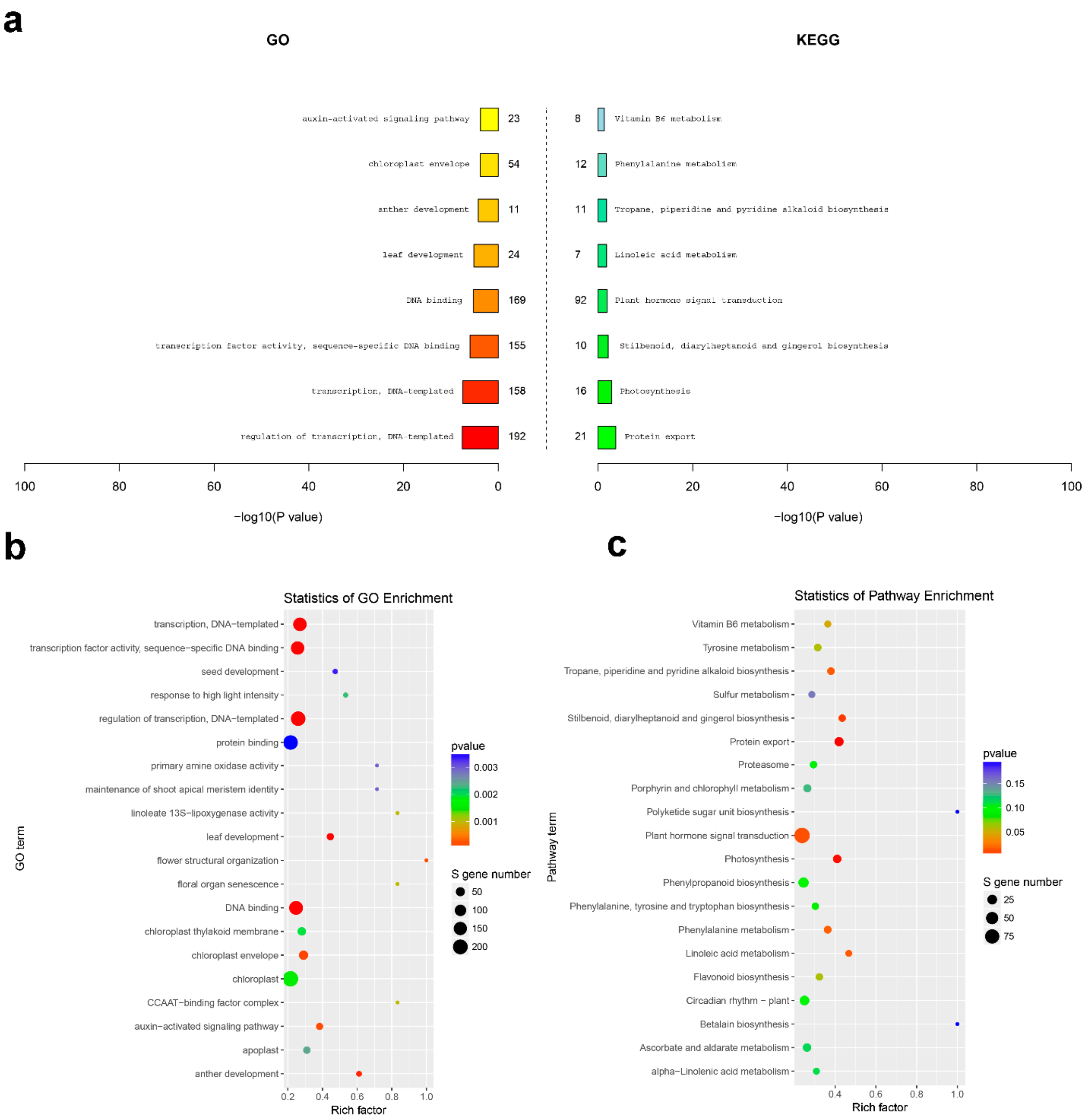

Supplementary Figure S4 Categories of conserved miRNAs based on their biological process activities

MIR319|MIR159|MIR399|	Gene silencing by miRNA
MIR168|MIR166|MIR165|	Posttranscriptional gene silencing by RNA
MIR395|MIR164|MIR161|	Gene silencing by RNA
MIR172|MIR160|MIR390|	Posttranscriptional gene silencing
MIR170	Gene silencing
	Posttranscriptional regulation of gene expression
	Regulation of gene expression, epigenetic
	Negative regulation of gene expression
	Negative regulation of macromolecule metabolic process
	Negative regulation of metabolic process
	Negative regulation of biological process
	Regulation of gene expression
	Regulation of macromolecule metabolic process
	Regulation of metabolic process
	Regulation of biological process
	Biological regulation
	Cellular process
MIR166|MIR164|MIR172|	Organ development
MIR319|MIR160	System development
	Anatomical structure development
	Multicellular organismal development
	Multicellular organismal process
	Developmental process
MIR164|MIR172|MIR160	Flower development
	Reproductive structure development
	Reproductive developmental process
MIR166|MIR164	MRNA cleavage involved in gene silencing by miRNA
	MRNA cleavage
	Nucleic acid phosphodiester bond hydrolysis
	MRNA processing
	MRNA metabolic process
MIR164|MIR160	Floral organ development
	Petal development
	Corolla development
	Floral whorl development
	Post-embryonic organ development
MIR399|MIR395	Cellular response to phosphate starvation
	Cell communication
	Cellular response to starvation
	Response to external stimulus
	Response to starvation
	Cellular response to nutrient levels
	Response to nutrient levels
	Cellular response to external stimulus
	Cellular response to extracellular stimulus
	Response to extracellular stimulus
MIR319|MIR160	Leaf development
	Shoot development
	Shoot system development
	Phyllome development
MIR166|MIR172	Tissue development
MIR159|MIR160	Response to abscisic acid stimulus
MIR159|MIR164	Negative regulation of cellular process
MIR159	Negative regulation of abscisic acid mediated signalin
	Regulation of abscisic acid mediated signaling pathwa
MIR164	Specification of petal number
	Specification of decreased petal number
	Specification of organ number
	Specification of floral organ number
	Petal formation
	Petal morphogenesis
	Floral organ formation
	Leaf senescence
	Organ senescence
	Floral organ morphogenesis
	Post-embryonic organ morphogenesis
MIR160	Sepal development
	Flower calyx development
	Regulation of anthocyanin biosynthetic process
	Regulation of anthocyanin metabolic process
	Regulation of flavonoid biosynthetic process
	Regulation of secondary metabolic process
	Auxin mediated signaling pathway
	Cellular response to auxin stimulus
MIR395	Cellular response to sulfur starvation
	Cellular response to sulfate starvation
	Cellular anion homeostasis
	Cellular di-, tri-valent inorganic anion homeostasis
	Cellular phosphate ion homeostasis
	Di-, tri-valent inorganic anion homeostasis
	Phosphate ion homeostasis
	Anion homeostasis
MIR172	Meristem determinacy
MIR408	Response to copper ion

Supplementary Figure S5 Clustering analysis of conserved miRNAs; f: families of miRNAs. Color intensity shows the stronger correlation.

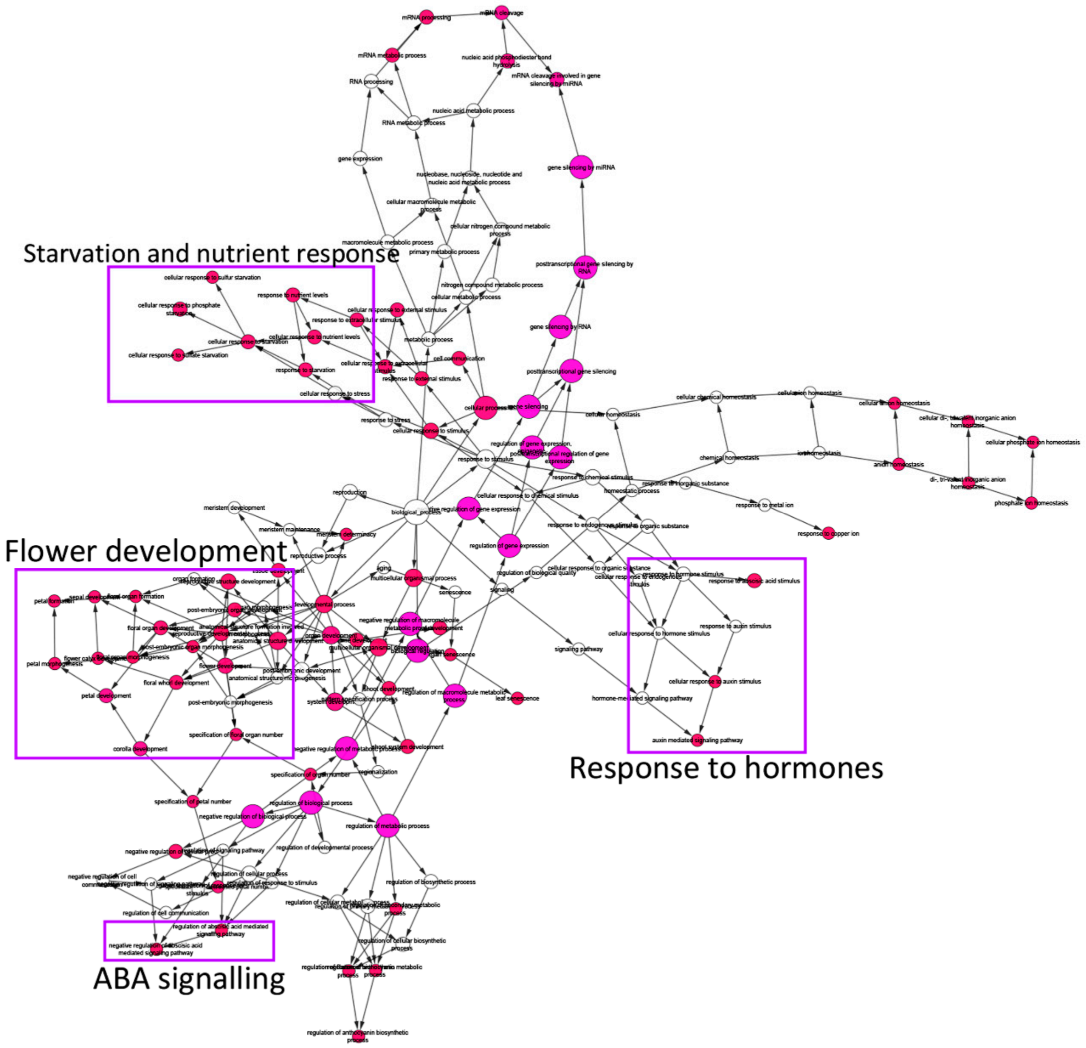

Supplementary Figure S6 Families of miRNAs.

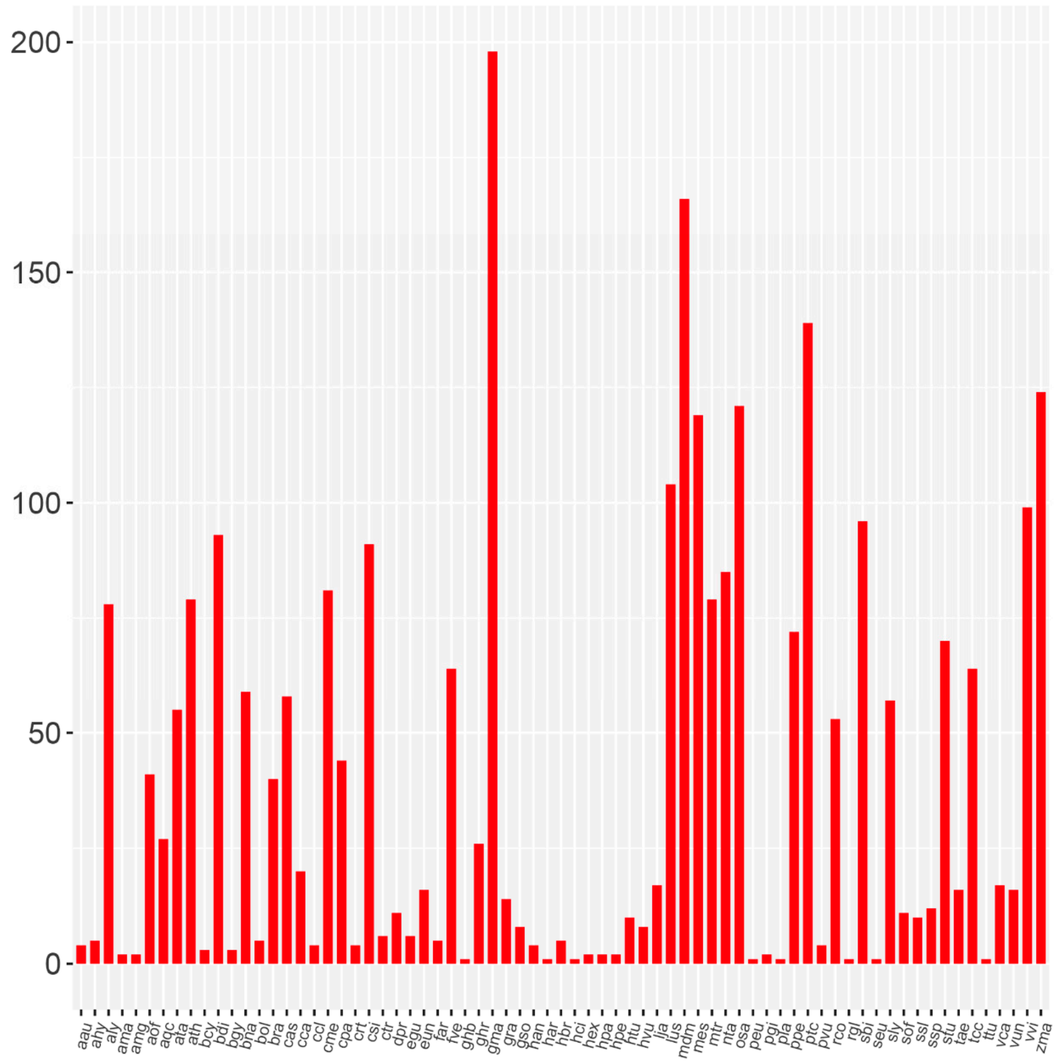

Supplement: Supplementary file 1 [file ijms-24-01699-s001.zip › Supplementary Figures.pdf]
